# Overview of systematic reviews of probiotics in the prevention and treatment of antibiotic-associated diarrhea in children

**DOI:** 10.3389/fphar.2023.1153070

**Published:** 2023-07-24

**Authors:** Qingrui Yang, Zeyu Hu, Yuyu Lei, Xinzhu Li, Chao Xu, Jie Zhang, Haitao Liu, Xiaoquan Du

**Affiliations:** ^1^ The First Clinical Medical College, Shaanxi University of Chinese Medicine, Xianyang, China; ^2^ Department of Gastroenterology, The Affiliated Hospital of Shaanxi University of Chinese Medicine, Xianyang, China

**Keywords:** children, antibiotic-associated diarrhea, probiotics, systematic review, overview of systematic reviews, PRISMA 2020, AMSTAR 2, grade

## Abstract

**Background:** Antibiotics alter the microbial balance commonly resulting in antibiotic-associated diarrhea (AAD). Probiotics may prevent and treat AAD by providing the gut barrier and restoring the gut microflora. This study will overview the Systematic Reviews (SRs) of probiotics in preventing and treating AAD in children. It will also assess the reporting, methodological, and evidence quality of the included SRs to provide evidence for their clinical practice.

**Methods:** After searching PubMed, Embase, Cochrane Library, CNKI, CBM, VIP, and WanFang Data databases, and finally included SRs of probiotics in the prevention and treatment of AAD in children, which were published before 1 October 2022. The reporting, methodological, and evidence quality of the included SRs were assessed by PRISMA 2020 statement, AMSTAR 2 tool, and GRADE system.

**Results:** A total of 20 SRs were included, and the results of PRISMA 2020 showed that 4 out of 20 SRs with relatively complete reporting, and the others within some reporting deficiencies, with scores ranging from 17 points to 26.5 points; the results of AMSTAR 2 showed that 3 SRs belonged to moderate quality level, 10 SRs belonged to low-quality level and 7 SRs being extremely low-quality level; the results of the GRADE system showed that a total of 47 outcomes were reported for the included SRs, three were high-level evidence quality, 16 were medium-level evidence quality, 24 were low-level evidence quality, and four were extremely low-level evidence quality; the results of the Meta-analysis showed that high doses (5–40 billion CFUs per day) of probiotics had a significant effect in the prevention of AAD, but it is too early to conclude the effectiveness and safety of other probiotic drugs for AAD in children, except for *Lacticaseibacillus rhamnosus* and *Saccharomyces boulardii.*

**Conclusion:** Current evidence shows that probiotics effectively prevent and treat AAD in children, and the effect of probiotics on pediatric AAD may be a potential dose-response effect. However, the conclusion should be treated with caution due to deficiencies in the methodological, reporting, and evidence quality of the included SRs. Therefore, the methodological, reporting, and evidence quality of relevant SRs still need further improvement.

**Systematic Review Registration:** https://www.crd.york.ac.uk/prospero/, identifier CRD42022362328

## 1 Introduction

Antibiotic-associated diarrhea (AAD) is defined as diarrhea that occurs in the long-term use of antimicrobial drugs leading to dysbiosis of the intestinal flora (Bartlett, 2002). With the increasing degree of intestinal dysbiosis, the clinical manifestations of AAD can progress from mild diarrhea to acute and severe disease such as pseudomembranous colitis or toxic megacolon (seen in *Clostridium difficile* infection) (Bartlett, 2002; Zheng et al., 2021). The incidence and severity of clinical manifestations of AAD are related to the type of antibiotic, duration of use, patient health status, and the type of pathogen to which the patient is exposed (McFarland, 2008; Hayes and Vargas, 2016). Some studies showed that the incidence of childhood AAD in the United States ranged from 6% in outpatients to 80% in hospitalized children (McFarland et al., 2016). The incidence of childhood AAD in China has only been studied in hospitalized children, with incidence rates ranging from 16.80% to 70.59% (Zheng et al., 2021).

Currently, antibiotic-induced dysbiosis of the intestinal flora is the primary mechanism of AAD pathogenesis, and the basic therapeutic approach is re-establishing intestinal flora homeostasis (Zheng et al., 2021). Clinical commonly used bioactive agents, such as probiotics (living microorganisms, when administered with sufficient amounts of probiotics, may bring health benefits to the host) (Hill et al., 2014), prebiotics (a substrate that is selectively utilized by the microorganisms of the host, conferring a health benefit) (Gibson et al., 2017), synbiotics (a mixture comprising live microorganisms and substrates selectively utilized by host microorganisms that confers a health benefit on the host) (Swanson et al., 2020), and postbiotics (preparation of inanimate microorganisms and their components that confers a health benefit on the host) (Salminen et al., 2021). The European Society for Paediatric Gastroenterology, Hepatology and Nutrition (ESPGHAN) recommendations for probiotics to prevent antibiotic-associated diarrhea high doses (≥5 billion CFU/day) of *Lacticaseibacillus rhamnosus* (*L. rhamnosus*) *GG* or *Saccharomyces boulardii* (*S. boulardii*) started simultaneously with antibiotic treatment (certainty of evidence: moderate; grade of recommendation: strong) (Szajewska et al., 2023) There are many systematic reviews (SRs) that have explored the efficacy and adverse effects of probiotics in pediatric AAD (Johnston et al., 2006; Szajewska et al., 2006; Johnston et al., 2011; Szajewska and Kołodziej, 2015a; Szajewska and Kołodziej, 2015b; Goldenberg et al., 2015; Szajewska et al., 2016; Xu et al., 2017; Guo et al., 2019; Storr and Stengel, 2021), however, their methodological, reporting and evidence quality of evidence are unclear. An overview of systematic reviews is a comprehensive approach that collects relevant systematic reviews of the treatment, etiology, diagnosis, and prognosis of the same disease or health problem (Lunny et al., 2017; Lunny et al., 2018). The principal objective of this overview was to clarify the benefits of probiotics for the prevention or treatment of AAD in children, which promotes evidence-based decision-making. Therefore, this study will overview SRs related to probiotics in preventing and treating AAD in children. It will also assess the methodological, reporting, and evidence quality of the included SRs to provide evidence for their clinical practice.

## 2 Methods

### 2.1 Project registration

This study was registered in the PROSPERO platform at the beginning of the project, ID: CRD42022362328.

### 2.2 Data sources

The databases of PubMed, Embase, Cochrane Library, Chinese Biomedical Literature Database (CBM), Chinese Journal Full Text Database (CNKI), Vipers Database (VIP), and WanFang Data Knowledge Service Platform (WanFang Data) were searched from their inception to 1 October 2022. The languages were limited to Chinese and English. The search terms included: probiotics, microecological agents, children, antibiotic associated diarrhea, systematic reviews, Mata analysis, Child, Antibiotic-associated diarrhea, Diarrhea, Systematic Review, and Meta-analysis. The specific search strategy for the PubMed database, for example, is shown in Table 1.

**TABLE 1:** PubMed retrieval strategy.

Process	Retrieval strategy
#1	“children”[MeSH Terms]
#2	“children*”[Title/Abstract] OR “pediatric*”[Title/Abstract] OR “toddler*”[Title/Abstract] OR “infant*”[Title/Abstract] OR “nurseling*”[Title/Abstract] OR “neonate*”[Title/Abstract]
#3	#1 OR #2
#4	“antibiotic-associated diarrhea”[Title/Abstract] OR “antibiotic associated diarrhea”[Title/Abstract] OR “AAD”[Title/Abstract]
#5	“probiotics”[MeSH Terms]
#6	“probiotic*”[Title/Abstract] OR “probiotic bacteria”[Title/Abstract] OR “beneficial bacteria”[Title/Abstract] OR “probiotic agent”[Title/Abstract] OR “probiotic preparation”[Title/Abstract] OR “microecological preparation”[Title/Abstract] OR “lactobacillus*”[Title/Abstract] OR “*streptococcus* thermophilus”[Title/Abstract] OR “bifidobacterium*”[Title/Abstract] OR “*clostridium* butyricum”[Title/Abstract] OR “saccharomyces*”[Title/Abstract] OR “bacillus*”[Title/Abstract]
#7	#5 OR #6
#8	“systematic review”[Publication Type] AND “systematic reviews as Topic”[MeSH Terms]
#9	“systematic review”[Title/Abstract] OR “systematic reviews”[Title/Abstract] OR “meta-analysis”[Title/Abstract] OR “mata analysis”[Title/Abstract] OR “meta analyses”[Title/Abstract]
#10	#8 OR #9
#11	#3 AND #4 AND #7 AND #10

### 2.3 Inclusion criteria

#### 2.3.1 Type of study

Systematic review or Meta-analysis.

#### 2.3.2 Study population

(1) Patient type: patients with AAD; (2) Referring to the definition of children in Pediatrics: children aged ≤18 years old (Pomerance, 1997). There was no restriction on their gender or duration of illness.

#### 2.3.3 Interventions

The treatment group was probiotics or probiotics combined with conventional Western medical treatment (CWM), and the control group was CWM, placebo, or blank control. The type, usage, dose, and duration of probiotics were not limited.

#### 2.3.4 Outcome indexes

Any efficacy and safety indexes.

### 2.4 Exclusion criteria

(1) Duplicate published literature; (2) Literature with inaccessible full text or incomplete data; (4) Studies containing a systematic review and Meta-analysis of other types of diarrhea; (3) Probiotic-related review studies.

### 2.5 Literature screening and data extraction

Two researchers (YL and XL) independently screened the literature and extracted data. They cross-checked them in parallel and negotiated, discussed, or consulted a third researcher (XD) in case of disagreement. Data extraction included: authors, disease names, sample size and interventions, and Meta-analysis results.

### 2.6 Quality assessment

Two researchers independently evaluated the reporting, methodological, and evidence quality of the included SRs using PRISMA 2020 (Page et al., 2021a; Page et al., 2021b), AMSTAR 2 (Shea et al., 2017) and the GRADE system (Atkins et al., 2004; Balshem et al., 2011), cross-checking in parallel and consulting a third party in case of disagreement. PRISMA 2020 consists of 27 items, and each item is scored as 1) fully satisfied (i.e., complete reporting) is scored as 1; 2) partially satisfied (i.e., partial reporting) is scored as 0.5; and 3) not satisfied (i.e., not reported) is scored as 0. AMSTAR 2 consists of 16 items, of which 7 are key items; each item is evaluated as “yes” (fully reported), “partially yes” (partially reported), and “partially yes” (partially reported). Combining the results of the key and non-key item assessments, each included SR was rated as high, moderate, low or very low in quality. Escalation factors for GRADE are large effect size, dose-effect relationship, and negative bias, and the downgrading factors are risk of bias, inconsistency, indirectness, imprecision, and publication bias. The level of evidence for the indicators was evaluated as high, moderate, low, or very low. Two researchers (YL and XL) independently assessed the evidence quality.

### 2.7 Statistical analysis

The extracted information was collated using Excel 2020—descriptive statistical analysis of frequency and percentage of the included studies. The risk ratio (RR), odds ratio (OR), 95% confidence interval (CI), weighted mean difference (WMD), standard mean difference (SMD), Relative Risk Reduction (RRR), and number needed to treat (NNT) were included to summarize the results. The heterogeneity of each included SR was extracted, which was detected by *I* ^*2*^ statistics.

## 3 Results

### 3.1 Literature search

**FIGURE 1:**
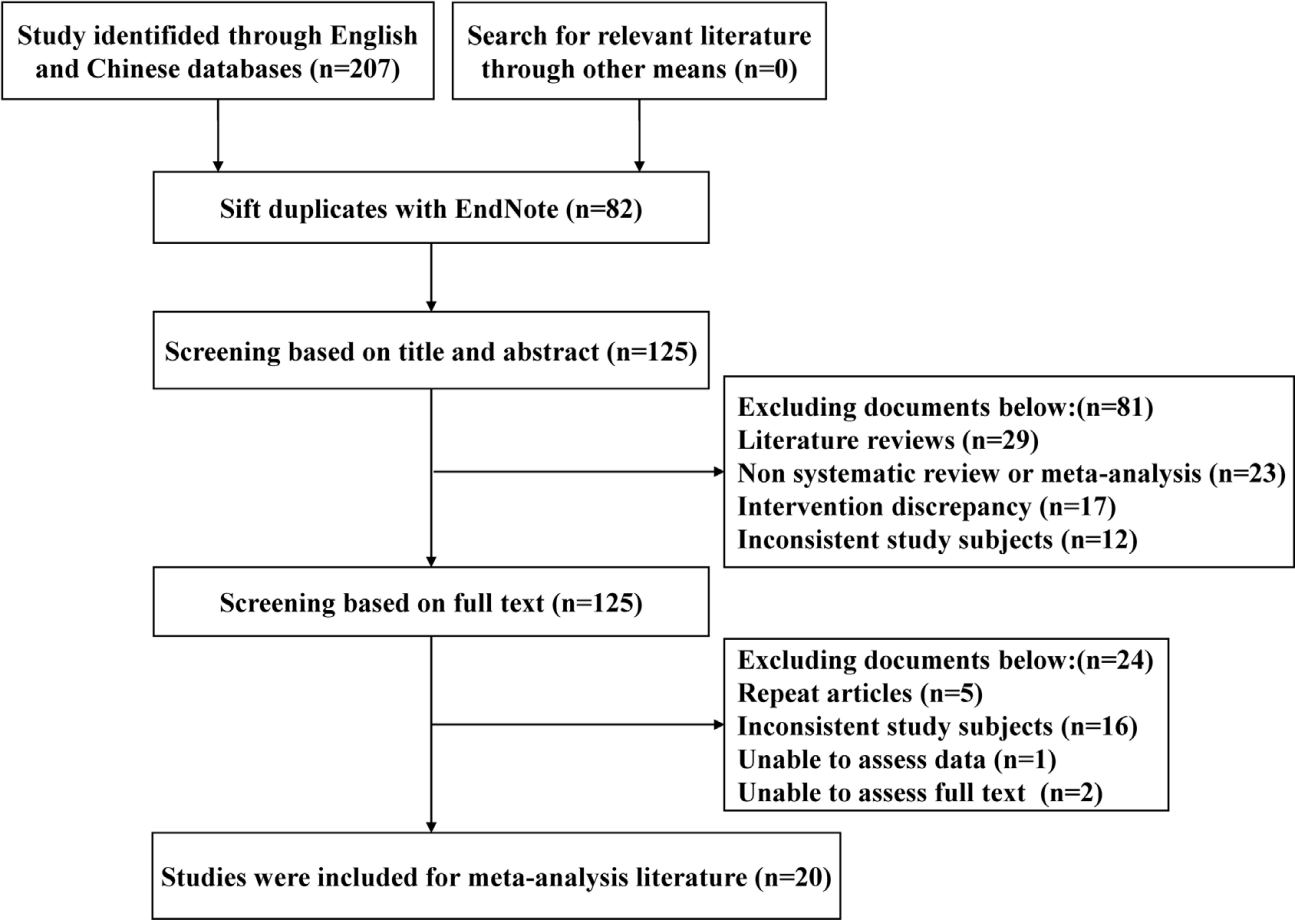
Flow chart of the study selection process.

A total of 207 studies were obtained for the initial review, and 20 SRs were finally included after a hierarchical screening process (Johnston et al., 2006; Shi et al., 2006; Szajewska et al., 2006; Chen et al., 2010; Lu, 2010; Johnston et al., 2011; Fang et al., 2013; Chai et al., 2015; Goldenberg et al., 2015; You and Gao, 2015; Yang et al., 2016a; Yang et al., 2016b; Szajewska et al., 2016; Zhou et al., 2016; Chai et al., 2017; He et al., 2017; Xu et al., 2017; Guo et al., 2019; Liu et al., 2020; Liu et al., 2022), and the literature screening process and results are shown in Figure 1.

### 3.2 Study characteristics

The basic information of the included studies is shown in Table 2. Among the 20 SRs included, 13 SRs (Shi et al., 2006; Chen et al., 2010; Lu, 2010; Fang et al., 2013; Chai et al., 2015; You and Gao, 2015; Yang et al., 2016a; Yang et al., 2016b; Zhou et al., 2016; Chai et al., 2017; He et al., 2017; Liu et al., 2020; Liu et al., 2022) were in Chinese and seven SRs (Johnston et al., 2006; Szajewska et al., 2006; Johnston et al., 2011; Goldenberg et al., 2015; Szajewska et al., 2016; Xu et al., 2017; Guo et al., 2019) were in English, published from 2006 to 2022. All SRs were included in randomized controlled trial studies (RCTs), and all used Meta-analysis to process the data. The main probiotics include *Bacillus* spp., *Bifidobacterium* spp., *Lacticaseibacillus* spp.*, Lactococcus spp.*, *Saccharomyces* spp.*, and Streptococcus spp*. The daily dosage of probiotics varied greatly from 1 million to 2 trillion CFUs/day. Twelve SRs (Szajewska et al., 2006; Chen et al., 2010; Johnston et al., 2011; Chai et al., 2015; Goldenberg et al., 2015; Yang et al., 2016a; Yang et al., 2016b; Szajewska et al., 2016; Chai et al., 2017; Guo et al., 2019; Liu et al., 2020; Liu et al., 2022) used the Cochrane systematic review tool, seven SRs (Johnston et al., 2006; Shi et al., 2006; Lu, 2010; Fang et al., 2013; Zhou et al., 2016; He et al., 2017; Xu et al., 2017) used the Jadad scale, and one SR (You and Gao, 2015) did not report a risk of the bias assessment tool.

**TABLE 2:** Basic characteristics of included systematic reviews.

Reviews (year)	RCT included (sample size)	Population	Age	Intervention measures (T/C)		Probiotic species	Probiotic dose (CFU/day)	RRR	NNT	Duration of diarrhea(d)	Quality assessment	Data analysis methods	Outcome indiccators
Liu et al. (2022)	29 (4096)	Infants and children	NR	Viable *Clostridium* butyricum and CWM	CWM	CB、BI	1–4 million	70%	6	−1.87d	Cochrane ROB	MA	①②③⑤
He et al. (2017)	8 (1880)	Children	NR	PP	Placebo	CB, BI, LR, BL, LP, ST, SB, LGG	1 million–20 billion	67%	9	−1.77d	Jadad	MA	①②③⑥⑦
Lu (2010)	9 (975)	Children	NR	PP	CWM	SB, LGG, CB, LA, LB, BL, LS	1 million–20 billion	NR	NR	NR	Jadad	MA	④
Shi et al. (2006)	5 (1560)	Newborn	≤28d	PP	Blank	BL, LA, EF, BS	1–135 million	67%	7	NR	Jadad	MA	①
Chen et al. (2010)	8 (1114)	Children	0–18 y	PP	Blank	LGG, LS, SB, BC, ST, BL, LR, LP	1 million—55 billion	64%	7	NR	Cochrane ROB	MA	①
Liu et al. (2020)	7 (768)	Children	NR	Bifidobacterium preparations and CWM	CWM	BL, LA, EF, BC, LB, ST	1–60 million	NR	NR	−1.67d	Cochrane ROB	MA	②③④⑤
Chai et al. (2017)	12 (1761)	Infants and children	≤3 y	Bifidobacterium tetravaccine tablets	CWM	BL, LA, EF, BC	1–3 million	NR	NR	NR	Cochrane ROB	MA	②④
You and Gao. (2015)	21 (3881)	Children	≤12 y	Live combined *Bacillus subtilis* and CWM	CWM	EF, BS	15–810 million	61%	5	NR	NK	MA	①②④⑤
Yang et al. (2016a)	23 (3939)	Children	≤18 y	*Saccharomyces* boulardii sachets and CWM	CWM	SB	162.5–650 million	54%	5	−1.82	Cochrane ROB	MA	①②③④⑤⑨
Yang et al. (2016b)	6 (746)	Children	0–18 y	*Saccharomyces* boulardii sachets and CWM	CWM	SB	162.5–650 million	NR	NR	−1.95	Cochrane ROB	MA	②③④⑧
Zhou et al. (2016)	12 (2750)	Children	<18 y	*Saccharomyces* boulardii sachets and CWM	CWM	SB	325–650 million	47%	7	−1.17	Jadad	MA	①③④⑤
Chai et al. (2015)	17 (2389)	Infants and children	≤3 y	*Saccharomyces* boulardii sachets	Blank	SB	325–650 million	55%	5	NR	Cochrane ROB	MA	①②
Fang et al. (2013)	9 (1511)	Children	<18 y	*Saccharomyces* boulardii sachets	Blank	SB	325–650 million	50%	6	NR	Jadad	MA	①②
Xu et al. (2017)	30 (7225)	Children	0–14 y	Bifidobacterium preparations	Blank	LA, BL, EF, LB, ST, BC, CB, BI	1–30 million	NR	NR	NR	Jadad	MA	①
Szajewska et al. (2016)	21 (3255)	Children	NR	PP	Placebo	LGG, SB, BC, PL, ST, LA, LB, BB, BL, LR, LP, BI	1 million—40 billion	57%	9	NR	Cochrane ROB	MA	①⑥
Szajewska et al. (2006)	6 (766)	Children	NR	PP	Placebo	LGG, LA, BI, LB, BL, ST, SB	1 million—20 billion	60%	7	NR	Cochrane ROB	MA	①③⑥
Johnston et al. (2011)	16 (3432)	Children	0–18 y	PP	Placebo	BA, BB, BC, BI, BL, LA, LB, LC, LGG, LP, LR, LS, SB, ST	200 million - 40 billion	51%	12	−1.18	Cochrane ROB	MA	①②③⑦
Johnston et al. (2006)	6 (707)	Children	0–18 y	PP	Placebo	LA, LB, LGG, SB, LS	3–40 billion	61%	6	NR	Jadad	MA	①②
Goldenberg et al. (2015)	23 (3938)	Children	0–18 y	PP	Placebo	BA, BB, BC, BI, BL, LA, LB, LC, LD, LGG, LL, LP, LR, LS, SB, SF, ST	100 million - 40 billion	57%	10	−0.6	Cochrane ROB	MA	①②③⑦
Guo et al. (2019)	33 (6352)	Children	0–18 y	PP	Placebo	BA, BB, BC, BI, BL, LA, LB, LC, LD, LGG, LL, LP, LR, LS, SB, SF, ST	100 million–2 trillion	58%	9	−0.91	Cochrane ROB	MA	①②③

NR, not reported; C, treatment group; T, control group; PP, probiotic preparations; CWM, conventional western medicine treatment; RRR, Relative Risk Reduction (percentage of reduction of AAD); NNT, number needed to treat; MA, Meta-Analysis.

Strain of probiotics: BA, Bififidobacteria anamalis subsp. lactus; BB, Bififidobacterium breve; BC, *Bacillus* clausii; BI, Bififidobacterium infantis; BL, Bififidobacterium lactis; BS, *Bacillus subtilis*; CB, *Clostridium* butyricum; EF, *Enterococcus faecalis*; LA, *Lactobacillus* acidophilus; LB, *Lactobacillus* bularicus; LC, Lactococcus casei; LD, *Lactobacillus* delbrueckii subsp. bulgaris; LGG, Lacticaseibacillus rhamnosus GG; LL, Lactococcus lactis; LP, Lactococcus plantarum; LR, Lactococcus rhamnosus; LS, *Lactobacillus* sporogens; SB, *Saccharomyces* boulardii; SF, *Saccharomyces* flflorentinus; ST, *Streptococcus* thermophilus.

*Outcomes:* ① *incidence of AAD;* ② *adverse effects;* ③ *duration of diarrhea;* ④ *total effective rate;* ⑤ *mean hospital stay;* ⑥ *incidence of CDAD;* ⑦ *mean stool frequency;* ⑧ *cure rate;* ⑨ *antidiarrheal time*.

### 3.3 Reporting quality

The results of the PRISMA 2020 are shown in Table 3: the scores of the included 20 SRs ranged from 16.5 to 26.5, four SRs (Johnston et al., 2006; Johnston et al., 2011; Goldenberg et al., 2015; Guo et al., 2019) (20%) had relatively complete reports, and 16 SRs (Shi et al., 2006; Szajewska et al., 2006; Chen et al., 2010; Lu, 2010; Fang et al., 2013; Chai et al., 2015; You and Gao, 2015; Yang et al., 2016a; Yang et al., 2016b; Szajewska et al., 2016; Zhou et al., 2016; Chai et al., 2017; He et al., 2017; Xu et al., 2017; Liu et al., 2020; Liu et al., 2022) (80%) had some reporting deficiencies. Among the reporting deficiencies, the main ones were found in item 24: Program and registration, followed by item 15: Other analysis in the methods section and item 22: Other analysis in the results section, as well as other information related to the item on funding.

**TABLE 3:** Quality of reporting of included systematic reviews assessed using the PRISMA 2020 statement.

Article structure	PRISMA 2020 item	Full report		Partial report		Unreported	
		Number, article	Percentage,%	Number, article	Percentage,%	Number, article	Percentage,%
Title	1. Title	20	100	0	0	0	0
Abstract	2. Structural Summary	3	15	17	85	0	0
Preface	3. Background	20	100	0	0	0	0
	4. Objective	20	100	0	0	0	0
Methods	5. Inclusion Criteria	20	100	0	0	0	0
	6. Information source	20	100	0	0	0	0
	7. Search	3	15	17	85	0	0
	8. Study Selection	18	90	1	5	1	5
	9. Data Extraction	18	90	1	5	1	5
	10. Data items	16	80	3	15	1	5
	11. Bias in individual studies	19	95	1	5	0	0
	12. Merger Effect Indicators	20	100	0	0	0	0
	13. Result Synthesis	13	65	7	35	0	0
	14. Study bias	20	100	0	0	0	0
	15. Other Analysis	4	20	0	0	16	80
Results	16. Study Selection	5	25	8	40	7	35
	17. Research Characteristics	18	90	2	10	0	0
	18. Risk of internal bias in research	19	95	0	0	1	5
	19. Individual study results	20	100	0	0	0	0
	20. Synthesis of results	16	80	4	20	0	0
	21. Inter-study bias	20	100	0	0	0	0
	22. Other Analysis	4	20	0	0	16	80
Discussion	23. Evidence Summary; Limitations; Conclusion	17	85	3	15	0	0
Other Information	24. Programs and Registration	3	15	0	0	17	85
	25. Funding	5	25	7	35	8	40
	26. Conflicts of interest	5	25	0	0	15	75
	27. Public information	0	0	20	100	0	0

### 3.4 Methodological quality

The results of the AMSTAR 2 are shown in Table 4: in the 20 SRs included, three SRs (Johnston et al., 2011; Goldenberg et al., 2015; Guo et al., 2019) (15%) were of medium quality, 10 SRs (Johnston et al., 2006; Szajewska et al., 2006; Chen et al., 2010; Fang et al., 2013; Yang et al., 2016a; Zhou et al., 2016; Chai et al., 2017; Xu et al., 2017; Liu et al., 2020; Liu et al., 2022) (50%) were of low quality, and seven SRs (Shi et al., 2006; Lu, 2010; Chai et al., 2015; You and Gao, 2015; Yang et al., 2016b; Szajewska et al., 2016; He et al., 2017) (35%) were of very low quality. The main reason for the lower quality level was that item 10, item 2, item 12, item 3, item 7, and item 16 were not reported.

**TABLE 4:** Methodological quality of included systematic reviews assessed using the AMSTAR 2 tool.

Reviews (year)	Item																Quality level
	1	2	3	4	5	6	7	8	9	10	11	12	13	14	15	16	
Liu et al. (2022)	Y	N	PY	Y	Y	Y	PY	Y	Y	N	Y	N	PY	N	PY	N	Low
He et al. (2017)	Y	N	PY	Y	Y	Y	N	Y	Y	N	Y	N	Y	PY	Y	Y	Critically low
Lu (2010)	Y	N	PY	Y	N	N	N	Y	Y	N	Y	N	N	Y	Y	N	Critically low
Shi et al. (2006)	Y	N	PY	Y	Y	Y	N	Y	Y	N	Y	N	PY	Y	Y	N	Critically low
Chen et al. (2010)	Y	N	PY	Y	Y	Y	Y	Y	Y	N	Y	N	Y	Y	Y	N	Low
Liu et al. (2020)	Y	N	PY	Y	Y	Y	PY	Y	Y	N	Y	N	Y	Y	Y	Y	Low
Chai et al. (2017)	Y	N	PY	PY	Y	Y	PY	Y	Y	N	Y	N	Y	Y	Y	Y	Low
You and Gao. (2015)	Y	N	PY	PY	Y	Y	N	PY	N	N	Y	N	Y	Y	PY	N	Critically low
Yang et al. (2016a)	Y	N	PY	Y	Y	Y	PY	Y	Y	N	Y	N	Y	Y	Y	Y	Low
Yang et al. (2016b)	Y	N	PY	Y	Y	Y	N	PY	Y	N	Y	N	Y	Y	Y	Y	Critically low
Zhou et al. (2016)	Y	N	PY	Y	Y	Y	PY	Y	Y	N	Y	N	Y	PY	Y	Y	Low
Chai et al. (2015)	Y	N	PY	Y	Y	Y	N	Y	Y	N	Y	N	Y	Y	Y	Y	Critically low
Fang et al. (2013)	Y	N	PY	Y	Y	Y	PY	Y	Y	N	Y	N	Y	Y	Y	N	Low
Xu et al. (2017)	Y	N	PY	Y	Y	Y	PY	Y	Y	N	Y	N	Y	N	Y	N	Low
Szajewska et al. (2016)	Y	N	Y	Y	PY	PY	N	Y	Y	Y	Y	N	Y	Y	N	Y	Critically low
Szajewska et al. (2006)	Y	N	Y	Y	Y	Y	Y	Y	Y	N	Y	N	Y	Y	Y	N	Low
Johnston et al. (2011)	Y	Y	Y	Y	Y	Y	Y	Y	Y	N	Y	Y	Y	Y	Y	Y	Moderate
Johnston et al. (2006)	Y	N	PY	Y	Y	Y	Y	Y	Y	N	Y	N	Y	Y	Y	Y	Low
Goldenberg et al. (2015)	Y	Y	Y	Y	Y	Y	Y	Y	Y	N	Y	Y	Y	Y	Y	Y	Moderate
Guo et al. (2019)	Y	Y	Y	Y	Y	Y	Y	Y	Y	N	Y	Y	Y	Y	Y	Y	Moderate

Y, yes; PY, Partially Yes: N, No.

### 3.5 GRADE quality of evidence

Based on the preventive and therapeutic effects of probiotics on ADD in clinical studies, as well as the adverse effects produced, the results of the quantitative analysis of the outcome indicators and the quality of evidence results of the 20 SRs from these three aspects are summarized and reported below, as detailed in Table 5.

**TABLE 5:** Qualities of the evidence measuring major outcomes rated by the GRADE system.

Reviews (year)	Outcome indiccator	Intervention measures (T/C)		Effect amount and 95%CI	Bias risk	Inconsistency	Indirectivity	Inaccuracy	Publication bias	Evidence quality
Liu et al. (2022)	①	Viable *Clostridium* butyricum and CWM	CWM	RR = 0.29, 95%CI [0.25, 0.34]	−1	0	0	0	0	Moderate✱
	③	Viable *Clostridium* butyricum and CWM	CWM	MD = −1.87, 95%CI [-2.11, −1.6]	−1	−1	0	0	0	Low✱$
	⑤	Viable *Clostridium* butyricum and CWM	CWM	MD = −1.96, 95%CI [-2.22, −1.70]	−1	−1	0	0	0	Low✱$
He et al. (2017)	①	PP	Placebo	RR = 0.32, 95%CI [0.24, 0.40]	0	0	0	0	0	High
	⑥	PP	Placebo	RR = 0.37, 95%CI [0.15, 0.91]	0	0	0	0	0	High
	③	PP	Placebo	MD = −1.77, 95%CI [-2.03, −1.51]	0	−1	0	0	0	Moderate$
	⑦	PP	Placebo	MD = −0.19, 95%CI [-0.38, −0.01]	0	−1	0	0	0	Moderate$
Lu. (2010)	④	PP	CWM	OR = 0.22, 95%CI [0.15, 0.32]	0	0	0	0	0	High
Shi et al. (2006)	①	PP	Blank	OR = 0.28, 95%CI [0.20, 0.38]	−1	0	0	0	−1	Low✱#
Chen et al. (2010)	①	PP	Blank	RR = 0.36, 95%CI [0.27, 0.48]	0	0	0	0	−1	Moderate#
Liu et al. (2020)	④	Bifidobacterium preparations and CWM	CWM	RR = 1.21, 95%CI [1.14, 1.27]	−1	0	0	0	−1	Low✱#
	③	Bifidobacterium preparations and CWM	CWM	SMD = −0.80, 95%CI [-1.05, −0.55]	−1	0	0	0	0	Moderate✱
	⑤	Bifidobacterium preparations and CWM	CWM	SMD = −0.49, 95%CI [-0.73, −0.25]	−1	0	0	0	0	Moderate✱
Chai et al. (2017)	④	Bifidobacterium tetravaccine tablets	CWM	OR = 5.74, 95%CI [4.14, 7.96]	0	0	0	0	−1	Moderate#
You and Gao. (2015)	①	Live combined *Bacillus subtilis* and CWM	CWM	OR = 0.27, 95%CI [0.22, 0.32]	−1	0	0	0	0	Moderate✱
	④	Live combined *Bacillus subtilis* and CWM	CWM	OR = 6.76, 95%CI [4.16, 10.98]	−1	0	0	0	0	Moderate✱
Yang et al. (2016a)	①	*Saccharomyces* boulardii sachets and CWM	CWM	RR = 0.47, 95%CI [0.42, 0.53]	−1	0	0	0	−1	Low✱#
	④	*Saccharomyces* boulardii sachets and CWM	CWM	RR = 1.34, 95%CI [1.22, 1.47]	−1	0	0	0	0	Moderate✱
	③	*Saccharomyces* boulardii sachets and CWM	CWM	MD = −1.82, 95%CI [-2.15, −1.48]	−1	−1	0	0	−1	Critically low✱$#
	⑨	*Saccharomyces* boulardii sachets and CWM	CWM	MD = −1.60, 95%CI [-1.71, −1.48]	−1	0	0	0	0	Moderate✱
	⑤	*Saccharomyces* boulardii sachets and CWM	CWM	MD = −2.47, 95%CI [-2.65, −2.29]	−1	0	0	0	0	Moderate✱
Yang et al. (2016a)	④	*Saccharomyces* boulardii sachets and CWM	CWM	RR = 1.21, 95%CI [1.08, 1.35]	−1	0	0	0	0	Moderate✱
	⑧	*Saccharomyces* boulardii sachets and CWM	CWM	RR = 1.81, 95%CI [1.48, 2.20]	−1	0	0	0	−1	Low✱#
	③	*Saccharomyces* boulardii sachets and CWM	CWM	MD = −1.95, 95%CI [-2.56, −1.34]	−1	−1	0	0	0	Low✱$
Zhou et al. (2016)	①	*Saccharomyces* boulardii sachets and CWM	CWM	RR = 0.54, 95%CI [0.47, 0.61]	−1	0	0	0	−1	Low✱#
	④	*Saccharomyces* boulardii sachets and CWM	CWM	RR = 1.41, 95%CI [1.28, 1.56]	−1	0	0	0	0	Moderate✱
	③	*Saccharomyces* boulardii sachets and CWM	CWM	SMD = −1.17, 95%CI [-1.48, −0.87]	−1	−1	0	0	0	Low✱$
	⑤	*Saccharomyces* boulardii sachets and CWM	CWM	SMD = −2.32, 95%CI [-4.05, −0.60]	−1	−1	0	0	0	Low✱$
Chai et al. (2015)	①	*Saccharomyces* boulardii sachets and CWM	CWM	OR = 0.32, 95%CI [0.27, 0.39]	−1	0	0	0	−1	Low✱#
Fang et al. (2013)	①	*Saccharomyces* boulardii sachets and CWM	CWM	RR = 0.49, 95%CI [0.41, 0.58]	−1	0	0	0	−1	Low✱#
Xu et al. (2017)	①	*Saccharomyces* boulardii sachets	Blank	OR = 0.33, 95%CI [0.29, 0.39]	−1	−1	0	0	0	Low✱$
Szajewska et al. (2016)	①	*Saccharomyces* boulardii sachets	Blank	OR = 0.48, 95%CI [0.37, 0.61]	−1	0	0	0	−1	Low✱#
	⑥	Bifidobacterium preparations	Blank	RR = 0.34, 95%CI [0.15, 0.76]	−1	0	0	0	0	Moderate✱
Szajewska et al. (2006)	①	PP	Placebo	RR = 0.44, 95%CI [0.25, 0.77]	−1	−1	0	0	−1	Critically low✱$#
	⑥	PP	Placebo	RR = 0.38, 95%CI [0.12, 1.18]	0	0	0	0	−1	Moderate#
Johnston et al. (2011)	①	PP	Placebo	RR = 0.52, 95%CI [0.38, 0.72]	−1	−1	0	0	0	Low✱$
	③	PP	Placebo	MD = −0.60, 95%CI [-1.18, −0.02]	−1	−1	0	0	0	Low✱$
	⑦	PP	Placebo	MD = −0.30, 95%CI [-0.60, −0.00]	−1	−1	0	0	0	Low✱$
	②	PP	Placebo	RD = 0.00, 95%CI [-0.01, 0.02]	−1	−1	0	0	−1	Critically low✱$#
Johnston et al. (2006)	①	PP	Placebo	RR = 0.43, 95%CI [0.25, 0.75]	−1	−1	0	0	0	Low✱$
Goldenberg et al. (2015)	①	PP	Placebo	RR = 0.46, 95%CI [0.35, 0.61]	−1	−1	0	0	0	Low✱$
	③	PP	Placebo	MD = −0.60, 95%CI [-1.18, −0.02]	−1	−1	0	0	0	Low✱$
	⑦	PP	Placebo	MD = −0.30, 95%CI [-0.60, 0.00]	−1	−1	0	0	0	Low✱$
	②	PP	Placebo	RD = 0.00, 95%CI [-0.01, 0.01]	−1	0	0	0	−1	Low✱#
Guo et al. (2019)	①	PP	Placebo	RR = 0.459, 95%CII [0.36, 0.56]	−1	−1	0	0	0	Low✱$
	③	PP	Placebo	MD = −0.91, 95%CI [-1.38, −0.44]	−1	−1	0	0	0	Low✱$
	②	PP	Placebo	RD = 0.00, 95%CI [-0.01, 0.01]	−1	−1	0	0	−1	Critically low✱$#

C, treatment group; T, control group; PP, probiotic preparations; CWM, conventional western medicine treatment.

*Outcomes:* ① *incidence of AAD;* ② *adverse effects;* ③ *duration of diarrhea;* ④ *total effective rate;* ⑤ *mean hospital stay;* ⑥ *incidence of CDAD;* ⑦ *mean stool frequency;* ⑧ *cure rate;* ⑨ *antidiarrheal time*.

OR, ratio; RR, relative risk; MD, weighted mean difference; SMD, standardized mean difference; RD, risk difference; −1: downgrade one level; 0: no downgrade; ✱: greater risk of bias in randomization, allocation concealment, and blinding; $: greater heterogeneity in combined results, I^2^ > 50%; #: potential for large publication bias.

#### 3.5.1 Indicators of preventive effects

##### 3.5.1.1 AAD incidence

Sixteen SRs (Johnston et al., 2006; Shi et al., 2006; Szajewska et al., 2006; Chen et al., 2010; Johnston et al., 2011; Fang et al., 2013; Chai et al., 2015; Goldenberg et al., 2015; You and Gao, 2015; Yang et al., 2016a; Szajewska et al., 2016; Zhou et al., 2016; He et al., 2017; Xu et al., 2017; Guo et al., 2019; Liu et al., 2022) reported the incidence of ADD. The GRADE system showed that one was of high quality, three were of moderate quality, eleven were of low quality, and one was of very low quality. The results showed that probiotics and probiotics combined with conventional western medical treatment were superior to conventional western medical treatment, placebo, and blank control in reducing the incidence of AAD, with statistically significant differences (*p* < 0.05).

##### 3.5.1.2 CDAD incidence

Three SRs (Szajewska et al., 2006; Szajewska et al., 2016; He et al., 2017) reported the incidence of *Clostridium* difficile-associated diarrhea (CDAD) belonging to severe AAD. The GRADE system showed that one was of high quality and two were of moderate quality, suggesting that probiotics were superior to placebo in the incidence of CDAD, with a statistically significant difference (*p* < 0.05).

#### 3.5.2 Indicators of treatment effects

##### 3.5.2.1 Duration of diarrhea

Duration of diarrhea was reported in 10 SRs (Szajewska et al., 2006; Johnston et al., 2011; Goldenberg et al., 2015; Yang et al., 2016a; Yang et al., 2016b; Zhou et al., 2016; He et al., 2017; Guo et al., 2019; Liu et al., 2020; Liu et al., 2022) and data were not combined for Meta-analysis in one SR (Szajewska et al., 2006). The GRADE system showed that two were of medium quality, six were of low quality, and one very low quality. Four of the SRs (Johnston et al., 2011; Goldenberg et al., 2015; He et al., 2017; Guo et al., 2019) with probiotics alone and five SRs (Yang et al., 2016a; Yang et al., 2016b; Zhou et al., 2016; Liu et al., 2020; Liu et al., 2022) by probiotics combined with conventional Western medical treatment showed superiority overview placebo and conventional Western medical treatment in reducing the duration of diarrhea, with statistically significant differences (*p* < 0.05).

##### 3.5.2.2 Total effective rate

Seven SRs (Lu, 2010; You and Gao, 2015; Yang et al., 2016a; Yang et al., 2016b; Zhou et al., 2016; Chai et al., 2017; Liu et al., 2020) mentioned the total effective rate. The GRADE system showed that one was high quality, five were medium quality, and one was low quality. The results suggest that the total effective rate of both probiotics and probiotics combined with conventional western medical treatment was better than conventional western medical treatment for AAD in children, and the difference was statistically significant (*p* < 0.05).

##### 3.5.2.3 Mean hospital stay

Five SRs (You and Gao, 2015; Yang et al., 2016a; Zhou et al., 2016; Liu et al., 2020; Liu et al., 2022) reported mean hospital stay, but one study (You and Gao, 2015) had a pooled data results of mean length of stay (MD: −53.19, 95% CI: −79.63 to −26.75), which was considered synthetically as a data error. Therefore, the quality of evidence was not evaluated for the outcome indicators in this overview. The GRADE system showed that two were medium quality and two were low quality. The results suggest that probiotic combined with conventional western medical treatment was superior to conventional western medical treatment in reducing the mean hospital stay in all cases, with a statistically significant difference (*p* < 0.05).

##### 3.5.2.4 Mean frequency of diarrhea

Three SRs (Johnston et al., 2011; Goldenberg et al., 2015; He et al., 2017) assessed the mean diarrhea frequency. The GRADE system showed that one was of medium quality and two were of low quality. The results of only one of these SRs (He et al., 2017) suggested a statistically significant difference in the mean frequency of diarrhea in children with AAD treated with probiotics compared with placebo (*p* < 0.05).

##### 3.5.2.5 Cure rate

Only one SRs (Yang et al., 2016b) analyzed the cure rate index. GRADE system the results as high quality, and the study showed that probiotics combined with conventional western medical treatment improved the cure rate of AAD in children compared with conventional western medical treatment alone, and the difference was statistically significant (*p* < 0.05).

##### 3.5.2.6 Antidiarrheal time

Only one SRs (Yang et al., 2016a) analyzed the time to stop diarrhea index, and the GRADE system the results as high quality. The study showed that using probiotics on top of conventional western medical treatment could be better than conventional western medical treatment in reducing the time to stop diarrhea, and the difference was statistically significant (*p* < 0.05).

#### 3.5.3 Adverse effects

Adverse Drug Reaction (ADR) was not defined in advance in all studies. 13 SRs (Johnston et al., 2006; Johnston et al., 2011; Fang et al., 2013; Chai et al., 2015; Goldenberg et al., 2015; You and Gao, 2015; Yang et al., 2016a; Yang et al., 2016b; Chai et al., 2017; He et al., 2017; Guo et al., 2019; Liu et al., 2020; Liu et al., 2022) reported ADRs, which mainly manifested as damage to the gastrointestinal digestive system and skin mucosa, including dry mouth, nausea, vomiting, belching, sputum, taste disturbance, loss of appetite, headache, chest pain, gastrointestinal distention, reflux, abdominal pain, constipation, rash, allergic reaction to antibiotics and mycosis stomatitis, but most studies did not report the group in which the ADR occurred (treatment or control group). There are six SRs (Johnston et al., 2006; Johnston et al., 2011; Goldenberg et al., 2015; Yang et al., 2016a; Guo et al., 2019; Liu et al., 2022) in the literature describing the specifics of ADR, of which only three SRs (Johnston et al., 2011; Goldenberg et al., 2015; Guo et al., 2019) combined data for Meta-analysis of ADR, and the GRADE system showed one of low quality and two of very low quality, which showed no statistically significant differences between the two groups (*p* > 0.05). In addition, two SRs (Szajewska et al., 2006; Szajewska et al., 2016) did not mention the specific occurrence of ADR, and five SRs (You and Gao, 2015; Yang et al., 2016b; Chai et al., 2017; He et al., 2017; Liu et al., 2020) mentioned that no ADR was seen. The above indicates that the incidence of ADRs in probiotics is low, suggesting that probiotics are safe to prevent and treat AAD.

## 4 Discussion

### 4.1 Major findings

The principle objective of this overview was to clarify the benefits of probiotics for preventing or treating AAD in children, which promotes evidence-based decision-making. The main used microorganisms in probiotic preparations in 20 SRs are bacteria of the *Lactobacillaceae* family, particularly *L. rhamnosus and L. acidophilus,* as well as *L. plantarum, L. case*i, *L. lactis* an*d L. bulgaricus*. Probiotics frequently contain bacteria of the genera *Bifidobacterium* (*B. longum, B. infantis, B. breve*), *Clostridium, Lactococcus, Enterococcus, Bacillus, and* strains of *S. thermophiles*. In addition, strains of *Saccharomyces* species, such as *S. boulardii* also present in these preparations (Table 2). We established some interesting findings through an in-depth review of the 20 studies.

Firstly, 16 studies reported the incidence of AAD, and five of them (Johnston et al., 2006; Johnston et al., 2011; Goldenberg et al., 2015; Xu et al., 2017; Guo et al., 2019) analyzed the incidence of AAD by intention-to-treat (ITT) analysis (overall patients as randomized were analyzed), results showed definite benefits of probiotics compared to active, placebo or no treatment controls. The pooled results of a per-protocol (PP) analysis (patients for whom data were available were analyzed as randomized) of one study (Xu et al., 2017) were similar to the ITT analysis (bifidobacterial preparations for the prevention or treatment of AAD in children). However, the ITT analysis was unreliable if the rate of lost to follow-up (LTFU) was high. Therefore, we chose the PP analysis for the pooled data results of the other four studies (Johnston et al., 2006; Johnston et al., 2011; Goldenberg et al., 2015; Guo et al., 2019). In addition, given that the definition of probiotics requires that “sufficient amounts” be given to achieve health benefits, it is unclear what the daily dose of probiotics should be. No dose-ranging studies have been reported to determine the minimum effective dose of probiotics in the prevention of AAD, and some studies (Ouwehand, 2017) suggest that doses near the lower range may not provide benefit, while doses in the higher range may be associated with an increased risk of adverse events. The daily doses of probiotics included in the 20 SRs were highly variable (1 million to 2 trillion CFU/d), with reductions in the incidence of AAD ranging from 47% to 70% after treatment with different probiotic dose interventions (corresponding probiotic doses of 325–650 million CPU/d for *S. boulardii* and 1-4 million CPU/d for *C. butyricum* and *B. infantis*) and a reduction in the duration of diarrhea of 0.6d–1.95 d (corresponding probiotic doses of *L. GG* 100 million-40 billion CPU/d and *S. boulardii* 162.5–650 million CPU/d) (Table 2). It suggests that the effect of probiotics on pediatric AAD may be a potential dose-response effect and that the use of probiotics during antibiotic use reduces the incidence of AAD. Notably, the SRs published in English are more in-depth than most published in Chinese regarding diarrhea incidence, especially in exploring the heterogeneity of the combined results. Several studies (Johnston et al., 2006; Johnston et al., 2011; Goldenberg et al., 2015; Guo et al., 2019) have critically evaluated each subgroup (e.g., probiotic type, probiotic dose, antibiotic class, and definition of diarrhea) by using multiple criteria. Subgroup analyses regarding probiotic dose compared low doses (<5 billion CFU/day) with high doses (≥5 billion CFU/day). For example, one study (Guo et al., 2019) reported a benefit of high-dose probiotics in AAD prevention, with a 63% reduction in the incidence of AAD with high-dose probiotics compared to controls (RR: 0.37, 95% CI: 0.30 to 0.46, *p* = 0.06, *I* ^2^ = 36%) and the NNT (i.e., number needed to treat) of 6 for prevention of one case of diarrhea (NNT: 6, 95% CI: 5–9).

### 4.2 Outcome indicators for systematic reviews

The included 20 SRs had some limitations in their analysis of outcome indicators. First, clinical efficacy may be affected because the effects of probiotics are strain-specific and dose-specific, and it is challenging to standardize specific interventions, doses, and regimens in clinical studies. For the preventive effect of probiotics, eight SRs (Johnston et al., 2006; Szajewska et al., 2006; Chen et al., 2010; Johnston et al., 2011; Goldenberg et al., 2015; Szajewska et al., 2016; Xu et al., 2017; Guo et al., 2019) have performed subgroup analyses of AAD incidence according to probiotic species, and the results suggest that it is too early to conclude the efficacy and safety of other probiotic drugs for AAD in children, except *L. rhamnosus* and *S. boulardii.* Four SRs (Johnston et al., 2006; Johnston et al., 2011; Goldenberg et al., 2015; Guo et al., 2019) performed subgroup analyses of AAD incidence according to probiotic dose subgroup analysis, with moderate quality evidence suggesting a significant role for high-dose (5–40 billion colony forming units per day) probiotics in the prevention of AAD. In addition, since multiple SRs were studying the same disease and data were collated and evaluated for the analysis of the same outcome indicators, there may be some overlap in the original studies included in different SRs. For example, two SRs (Johnston et al., 2011; Goldenberg et al., 2015) had the same Meta-analysis results for two outcome indicators (Table 5). On the other hand, the naming of the outcome indicators included in the SRs is highly variable, irregular, and even contradictory. Using of outcome indicators with different definition criteria may potentially affect the credibility of the conclusions. Therefore, there is a need to further promote the development of Core Outcome Set (COS) studies in the future, intending to address the problems of arbitrariness, inconsistency, and lack of recognition of clinical research outcome indicators (Williamson et al., 2012; Zhang et al., 2021).

### 4.3 Reporting quality of systematic reviews

According to the results of PRISMA 2020, certain reporting deficiencies exist: ① 85% of SRs failed to fully meet the requirements of structured abstracts, especially the study protocols and registration numbers of the original studies were not reported, which affected the reliability and rigour of the results; ② 85% of SRs only reported the search strategies of some databases, which affected the reproducibility of the results; ③ in terms of result synthesis, 35% of the SRs were not fully reported on data synthesis, mainly reflecting the lack of detailed and transparent methodological analysis of result heterogeneity and stability, which may potentially affect the reliability of the results; ④ 80% of SRs did not report content related to the strength of evidence for outcome indicators; ⑤ 35% of SRs did not provide a flow chart of the literature screening process, and 75% did not provide a list of excluded literature, which would affect the transparency and reproducibility of SRs production; ⑥ In terms of financial support, 40% of SRs did not report the source of funding for the study, while 75% of SRs did not report the role of the funder in completing the study, which could potentially have a conflict of interest and thus affect the study results; ⑦ All SRs did not fully disclose details about the data processing, which affected the recalibration and use of these data. In general, there is much room for future improvement in the standardization and rigour of report writing.

### 4.4 Methodological quality of systematic reviews

According to the results of AMSTAR 2, the deficiencies of key item 2 (reported the predefined protocol) and item 7 (List of excluded studies and reason) were found to be more obvious: ① 85% of the SRs did not provide a pre-study design plan, which would affect the rigour of the study results; ② 70% of the SRs did not provide a list of excluded literature in the screening process, which might have literature inclusion bias. In addition, the results of nine non-critical item assessments showed that: ① 75% of SRs did not describe the basis of study design selection (item 3), which may prevent a complete efficacy assessment of a certain intervention due to the type of study design included; ② 95% of SRs did not give information on the source of funds for inclusion in the original study (item 10), and 40% of SRs did not report potential conflicts of interest (item 16), which may affect the credibility of evidence-based conclusions; ③ 85% of SRs did not evaluate the impact of individual study risk of bias on the results of Meta-analysis (item 12), and inadequate assessment of the risk of bias may lead to biased results. Therefore, the methodological quality of relevant SRs still needs to be improved.

### 4.5 Quality of evidence for systematic reviews

The results of the GRADE system showed that high-quality evidence accounted for 6.25%, moderate-quality evidence for 33.33%, low-quality evidence for 52.08%, and very low-quality evidence for 8.33%. Major downgrading factors exist for risk of bias, inconsistency and publication bias. There is the unreasonable or incorrect implementation of random grouping, allocation concealment and blind implementation in the methodology design; the inconsistency is mainly reflected in large heterogeneity and low interval overlap; publication bias is reflected in small studies, funnel plot asymmetry or Egger’s test. In summary, the included studies were positive for the efficacy of probiotics in treating AAD in children, but the quality of evidence was generally low.

### 4.6 Study limitations

(1) A comprehensive literature search was conducted for this study, but due to language limitations, only Chinese and English SRs were included, which may be subject to potential publication bias. (2) The methodological, report, and evidence quality of the included SRs have certain shortcomings. There may be subjectivity in the study process, which reduces the reliability of the study results.

## 5 Conclusion

Overviews, as a comprehensive and relatively novel research method, assess the evidence from systematic reviews at a higher level, contain a richer and more comprehensive amount of information and can provide more focused evidence support for clinical researchers (McKenzie and Brennan, 2017; López-López et al., 2022). A total of 20 SRs were included in this study, which comprehensively compared the efficacy of probiotics in preventing and treating AAD in children. The results showed that probiotics alone or probiotics combined with conventional western medical treatment could not only effectively prevent the incidence of AAD and CDAD, but also improve the overall efficiency and clinical cure rate, shorten the duration of diarrhea, mean frequency of diarrhea, the average hospitalization time and antidiarrheal time, and the incidence of adverse effects was low, the safety of probiotics was good. However, the results of existing evidence show that the methodological, reporting and evidence quality of SRs of probiotics for AAD in children are generally low. There is still a need to improve the quality of evidence-based evidence to better explain the clinical application value of probiotics for AAD in children in the future. The results of this study need to be applied with reasonable interpretation.

## Data Availability

The raw data supporting the conclusion of this article will be made available by the authors, without undue reservation.
